# Adsorption-driven Graphene Oxide SPR Biointerfaces: Coupled Thermodynamic, Kinetic, and Optical Modeling with Effective-layer Validation

**DOI:** 10.1007/s12013-026-02105-0

**Published:** 2026-06-25

**Authors:** Sadok Kouz

**Affiliations:** Laboratoire Lumière Matière et Interfaces (LUMIN), UMR 9024, Ecole Normale Supérieure Paris-Saclay, CentraleSupélec, CNRS, Université Paris-Saclay, 4 avenue des Sciences, 91190 Gif-sur-Yvette, France

**Keywords:** Graphene oxide biointerface, Adsorption-driven sensing, Surface plasmon resonance, Langmuir–Hill adsorption, Binding kinetics, Transfer matrix modeling

## Abstract

This work presents a theoretical modeling framework for adsorption-driven graphene oxide (GO)-functionalized surface plasmon resonance (SPR) biointerfaces. Rather than describing a specific capture-probe biosensor, the model focuses on the effective optical transduction of molecular accumulation at a GO-modified interface. The framework couples transfer-matrix optical modeling of a prism/TiW/Au/GO multilayer structure with a generalized Langmuir–Hill adsorption formalism, allowing analyte concentration, surface coverage, effective interfacial refractive index, and plasmonic resonance shift to be linked within a unified description. The optical response is evaluated at 633 nm under angular interrogation. Adsorption is represented through effective surface coverage, an optically equivalent biolayer thickness, and a concentration-dependent refractive-index perturbation. Temperature dependence is incorporated using a van’t Hoff-type treatment for enthalpy-driven adsorption, while time-dependent behavior is described using first-order association–dissociation kinetics as an ideal reversible limiting case. Three representative adsorption regimes are considered, corresponding to weak, moderate, and cooperative surface accumulation, with equivalent dissociation constants ranging from approximately 1.67 to 200 *μ*M. A formal optical optimization identifies Au, TiW, and GO effective thicknesses of 49 nm, 0.20 nm, and 0.20 nm, respectively, producing a deep SPR minimum and a narrow angular linewidth. Because the TiW and GO optima occur at the lower boundary of the optical-thickness scan, they are interpreted as effective interfacial parameters rather than literal physical monolayer thicknesses. Additional simulations using physically realistic GO thicknesses of 0.7–1.5 nm show that the adsorption-driven response is preserved, with low-concentration sensitivities remaining close to 0.037–0.038 deg *μ*M^−1^. For the moderate adsorption regime, the model predicts a resonance shift of approximately 0.79^∘^ over 0–100 *μ*M, an effective biolayer thickness up to 2.72 nm, and a refractive-index sensitivity of 115.96 deg RIU^−1^. The estimated detection limit is shown to depend directly on the assumed angular noise. For an angular resolution of 10^−3^ degree, the model-estimated limit of detection is 0.027*μ*M, while varying the angular noise from 10^−4^ to 10^−2^ degree gives limits from 0.0027 to 0.270*μ*M. The temperature response is consistent with an exothermic adsorption case with *ΔH* ≈ − 26 kJ mol^−1^, and the kinetic analysis provides characteristic association and dissociation times under the assumed single-rate model. The results establish a mechanism-aware computational framework for connecting adsorption thermodynamics, kinetic parameters, and SPR optical response at GO biointerfaces, while clearly identifying the effective-parameter and experimental-validation limits of the model.

## Introduction

The quantitative and label-free analysis of molecular accumulation and interaction processes at interfaces remains a central challenge in modern biosensing, with important implications for diagnostics, molecular analysis, and biointerface engineering. In particular, the ability to monitor adsorption-driven processes in real time and without labeling is critical for understanding binding affinity, cooperativity, and surface-mediated phenomena in complex environments. In the context of graphene oxide (GO)-modified surfaces, such processes may include nonspecific adsorption, semi-specific surface affinity, molecular preconcentration, hydration-related interfacial changes, and effective adsorbed-layer formation, rather than only highly specific capture-probe recognition.

Surface plasmon resonance (SPR) sensors have emerged as powerful platforms for label-free and real-time detection of chemical and biological species [1–3]. Their operation relies on the excitation of surface plasmon polaritons at metal–dielectric interfaces, typically implemented in the Kretschmann configuration [4, 5]. Under resonance conditions, small variations in the refractive index near the sensing interface induce measurable shifts in the resonance angle, enabling highly sensitive probing of interfacial processes [6–8].

Despite these advantages, conventional SPR sensors based on single metallic layers exhibit intrinsic limitations, including relatively broad resonance features, reduced detection accuracy, and limited sensitivity in low-concentration regimes [9–11]. To overcome these challenges, multilayer architectures and engineered nanostructures have been extensively explored to enhance field confinement and improve plasmonic coupling efficiency [12–14].

In recent years, the integration of two-dimensional (2D) materials has provided new opportunities for improving SPR performance. Materials such as graphene, graphene oxide (GO), transition metal dichalcogenides (TMDs), and MXenes exhibit unique optical and electronic properties that enhance light–matter interaction at the sensing interface [15–18]. Among these, graphene oxide is particularly attractive due to its high surface area, tunable optical response, and strong molecular adsorption capability, making it highly suitable for biointerface engineering [19–21]. These properties enable enhanced analyte binding and more pronounced modulation of the local refractive index, thereby improving sensing performance [22–24]. However, because GO surfaces are chemically heterogeneous and rich in oxygen-containing functional groups, the resulting signal should often be interpreted as an adsorption-driven interfacial response unless a specific capture probe, receptor, antibody, aptamer, ligand, or DNA sequence is explicitly introduced. This distinction is important for defining the scope of theoretical GO-SPR models.

SPR-based platforms have been widely applied in the detection of biomolecules, pathogens, and chemical species [25–29]. In recent years, significant efforts have been devoted to enhancing sensing performance through advanced photonic architectures, including graphene-assisted multilayer systems and engineered resonant structures [30]. These approaches improve sensitivity and detection limits by strengthening light–matter interactions at the sensing interface. However, a key limitation persists in many theoretical models: the analyte is often represented solely through an effective refractive index change, without explicitly accounting for the underlying adsorption mechanisms that govern molecular binding at the interface.

Recent studies have demonstrated graphene oxide-enhanced multilayer SPR platforms for ultrasensitive molecular detection, including a five-layer GO/TiW/Au configuration analyzed using transfer matrix modeling and Langmuir-type adsorption isotherms [31]. While these approaches successfully improve sensor performance and capture adsorption-induced refractive index changes, they generally rely on simplified representations of molecular binding and are often restricted to single-analyte scenarios.

As a result, important biophysical aspects such as cooperative binding effects, thermodynamic behavior, and kinetic regimes remain insufficiently explored within a unified modeling framework. In addition, the physical interpretation of effective interfacial parameters, such as GO optical thickness, effective biolayer thickness, and model-derived detection limits, is not always explicitly separated from experimentally measurable quantities. This can lead to ambiguity when formal optical optimization yields subnanometer layer parameters or when idealized adsorption models are used to represent complex GO biointerfaces.

In realistic biointerfaces, the sensor response is fundamentally driven by adsorption thermodynamics and kinetics, which determine surface coverage, interfacial layer formation, and ultimately the optical perturbation sensed by the plasmonic field. Neglecting these processes limits both the physical realism and the predictive capability of conventional SPR models.

In this work, we address this limitation by developing a theoretical framework for an adsorption-driven graphene oxide-functionalized multilayer SPR biointerface. The proposed structure consists of a high-index prism, an ultrathin TiW adhesion layer, a gold plasmonic film, and a GO sensing layer. The optical response is modeled using the transfer matrix method, while interfacial molecular accumulation is described using a generalized Langmuir–Hill adsorption model that captures concentration dependence, cooperativity, and surface saturation effects.

This coupled approach enables a direct relationship between analyte concentration, surface coverage, and optical response, allowing for a realistic evaluation of sensing performance. In addition to concentration-dependent analysis, the framework incorporates temperature-dependent binding through thermodynamic modeling and time-dependent behavior through kinetic rate equations. The model is not intended to represent a complete capture-probe biosensor such as antibody–antigen recognition, aptamer–target binding, ligand–receptor binding, or DNA hybridization. Instead, it describes an effective GO-mediated adsorption process in which analyte concentration determines surface coverage, surface coverage modifies the effective refractive index and optically equivalent biolayer thickness, and these interfacial changes are transduced into an SPR resonance shift. The framework is therefore most directly applicable to adsorption-dominated GO biointerfaces, GO-mediated molecular preconcentration, and calibrated effective descriptions of interfacial accumulation.

A further objective of the revised framework is to clarify the physical interpretation of the effective layer parameters. In particular, subnanometer TiW and GO values obtained from formal optical optimization are treated as effective optical thicknesses rather than literal physical monolayer thicknesses. This distinction is important because realistic GO monolayers typically have larger physical thicknesses. Accordingly, additional simulations are included to compare the formal optical optimum with physically realistic GO thicknesses in the range 0.7–1.5 nm. Similarly, the modeled biolayer thickness is interpreted as an optically equivalent hydrated adlayer rather than as the geometric size of a specified molecule.

The central question addressed in this study is therefore: *how can adsorption-governed surface coverage at a GO-modified interface be quantitatively linked to thermodynamic parameters, kinetic response, effective interfacial optical properties, and SPR resonance shifts?*

By answering this question, this work establishes a mechanism-aware computational framework for GO-SPR biointerfaces while explicitly defining its assumptions, effective-parameter interpretation, and experimental-validation limits.

## Theoretical Model and Sensing Methodology

### GO-SPR Biointerface Architecture

The modeled platform is an adsorption-driven multilayer SPR biointerface based on a Kretschmann configuration. It is composed of a high-index prism, an ultrathin TiW adhesion layer, a gold plasmonic film, a graphene oxide (GO) interfacial layer, and an analyte-containing aqueous sensing medium. A p-polarized monochromatic beam at *λ* = 633 nm is incident through the prism, and the SPR condition is identified from the angular reflectance minimum.

In this framework, the GO layer is not treated as a specific capture-probe layer. Instead, it acts as an adsorption-active interfacial modifier that can promote molecular accumulation through its chemically heterogeneous surface, oxygen-containing functional groups, and interaction with the evanescent plasmonic field. Therefore, the modeled response corresponds to an effective adsorption-driven biointerface rather than a complete antibody–antigen, aptamer–target, ligand–receptor, or DNA-hybridization biosensor.

**Fig. 1:**
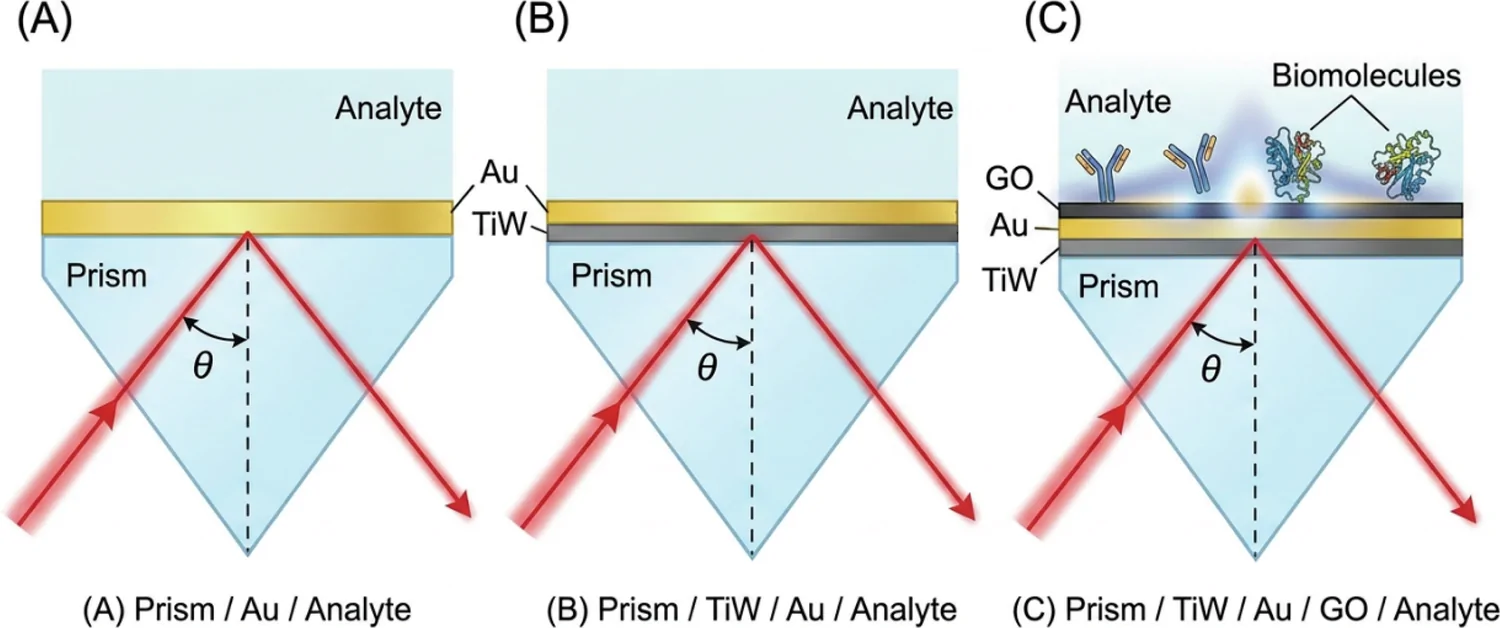
Comparison of three multilayer SPR configurations in Kretschmann geometry: (**A**) Prism/Au/Analyte, (**B**) Prism/TiW/Au/Analyte, and (**C**) Prism/TiW/Au/GO/Analyte. The progressive addition of TiW and GO layers enhances plasmonic coupling and introduces an adsorption-active GO interface. In configuration (**C**), molecular accumulation at the GO-modified surface interacts with the evanescent plasmonic field and is represented through an effective surface-coverage model

The TiW layer ensures mechanical stability and adhesion between the prism and the gold film, while the gold layer supports the excitation of surface plasmons. The GO overlayer modifies the optical boundary condition and provides an adsorption-active surface where molecular accumulation can perturb the effective interfacial refractive index and optically equivalent adlayer thickness. A schematic representation of the proposed sensing platform is shown in Fig. 1.

To isolate the role of each interfacial layer, three configurations are considered:

- Structure A: Prism/Au/Analyte
- Structure B: Prism/TiW/Au/Analyte
- Structure C: Prism/TiW/Au/GO/Analyte

### Transfer Matrix Formulation

The angular reflectance is calculated using the transfer matrix method (TMM) for p-polarized light, using the optical and model parameters summarized in Table 1. The in-plane wave vector is

1 $${k}_{x}={k}_{0}{n}_{0}\sin \theta ,\quad {k}_{0}=\frac{2\pi }{\lambda }.$$

For each layer *j*:

2 $${k}_{z,j}=\sqrt{{k}_{0}^{2}{\varepsilon }_{j}-{k}_{x}^{2}},\quad {q}_{j}=\frac{{k}_{z,j}}{{\varepsilon }_{j}}.$$

The characteristic matrix is

3 $${M}_{j}=\left[\begin{array}{cc}\cos {\delta }_{j}&-i\sin {\delta }_{j}/{q}_{j}\\ -i{q}_{j}\sin {\delta }_{j}&\cos {\delta }_{j}\end{array}\right],\quad {\delta }_{j}={k}_{z,j}{d}_{j}.$$

The total matrix is

4 $$M=\prod _{j}{M}_{j},$$

and the reflection coefficient is

5 $${r}_{p}=\frac{{q}_{0}{M}_{11}+{q}_{0}{q}_{s}{M}_{12}-{M}_{21}-{q}_{s}{M}_{22}}{{q}_{0}{M}_{11}+{q}_{0}{q}_{s}{M}_{12}+{M}_{21}+{q}_{s}{M}_{22}}.$$

The reflectance is *R*_*p*_ = ∣*r*_*p*_∣^2^, and the resonance angle ${\theta }_{{\rm{res}}}$ is extracted from the reflectance minimum using interpolation. Throughout this work, the TMM layer thicknesses should be interpreted as optical-model parameters. In particular, when formal optimization identifies subnanometer TiW or GO values, these values represent effective optical thicknesses within the multilayer calculation and should not automatically be interpreted as literal continuous physical monolayers. This point is examined explicitly in the realistic-thickness validation presented in the Results section.

**Table 1:** Optical and model parameters used in the transfer-matrix and adsorption simulations

Parameter	Value used	Role in the model
Interrogation wavelength	633 nm	Angular SPR interrogation wavelength
Angular scan window	40–89.9^∘^	Range used to locate the SPR minimum
Prism refractive index	1.723	Incident medium in Kretschmann geometry
Baseline buffer refractive index	1.3320	Aqueous sensing medium before adsorption
TiW complex refractive index	3.500 + 2.360*i*	Adhesion/effective interfacial layer
Au complex refractive index	0.170 + 3.420*i*	Plasmonic metal film
GO complex refractive index	1.800 + 0.020*i*	Adsorption-active interfacial optical layer
Baseline TiW/Au/GO thicknesses	0.50/46.00/0.50 nm	Initial stack before optimization
Optimized Au thickness	49.00 nm	Optimized plasmonic film thickness
Formal optimized TiW thickness	0.20 nm	Effective optical lower-bound adhesion layer
Formal optimized GO thickness	0.20 nm	Effective optical lower-bound GO layer
Realistic GO validation range	0.70–1.50 nm	Physical-realism check for GO thickness
Main adsorption affinity *K*_25_	8.0 × 10^4^ M^−1^	Moderate adsorption regime at 25 ^∘^C
Main Hill coefficient *n*	1.10	Weakly cooperative adsorption response
Adsorption enthalpy Δ*H*	− 26 kJ mol^−1^	Enthalpy-driven exothermic adsorption case
Association rate *k*_on_	1.2 × 10^4^ M^−1^ s^−1^	Effective single-rate kinetic model
Dissociation rate *k*_off_	1.5 × 10^−1^ s^−1^	Effective single-rate kinetic model
Maximum effective adlayer thickness ${d}_{\max }$	3.0 nm	Moderate-regime optically equivalent hydrated adlayer limit
Maximum refractive-index perturbation ${\rm{\Delta }}{n}_{\max }$	0.0070	Coverage-dependent effective sensing-region perturbation
Assumed angular resolution	10^−3^ degree	Reference value for theoretical LOD estimation

The optical constants are the numerical inputs used in the TMM calculation at 633 nm, while the adsorption parameters define the representative moderate adsorption regime used as the main case

### Adsorption-driven Biointerface Model

To connect analyte concentration to SPR response, adsorption at the GO-modified interface is described using a generalized Langmuir–Hill formalism. The model does not assign the analyte to a specific biomolecule or capture-probe pair. Instead, it represents parametric adsorption regimes at the GO interface, where concentration-dependent surface accumulation produces an effective optical perturbation.

The surface coverage is written as

6 $$\Theta (C)=\frac{{(KC)}^{n}}{1+{(KC)}^{n}},$$

where *C* is the analyte concentration, *K* is the effective affinity constant, and *n* is the Hill coefficient accounting for cooperative or non-cooperative adsorption behavior. For *n* ≈ 1, the model reduces to a Langmuir-like non-cooperative adsorption limit, whereas *n* > 1 represents a phenomenological cooperative surface-accumulation regime.

The adsorption-induced optical perturbation is modeled through two coupled effective quantities:

7 $${n}_{{\rm{eff}}}={n}_{{\rm{buffer}}}+\Delta {n}_{\max }\Theta ,$$

8 $${d}_{{\rm{bio}}}={d}_{\max }\Theta .$$

Here, *n*_eff_ is the effective refractive index of the sensing region and *d*_bio_ is the optically equivalent biolayer/adlayer thickness. The quantity *d*_bio_ should not be interpreted as the geometric diameter or physical height of a specified analyte molecule. It represents an effective hydrated optical adlayer that averages over molecular size, surface coverage, orientation, packing density, interfacial water, and dielectric contrast. Similarly, *n*_eff_ represents the effective refractive-index perturbation sensed by the evanescent plasmonic field rather than the bulk refractive index of a pure analyte solution.

This formulation establishes the following model chain:

$$C\to \Theta \to ({n}_{{\rm{eff}}},{d}_{{\rm{bio}}})\to \,\text{SPR response}\,.$$

This chain is the central effective-parameter assumption of the model: concentration determines surface coverage, surface coverage determines the optical perturbation of the GO interface, and the modified optical boundary condition determines the SPR resonance shift. The model is therefore best understood as a mechanism-aware adsorption-transduction framework rather than an experimentally calibrated description of a specific molecular-recognition assay.

### Thermodynamic Model

The temperature dependence of the effective adsorption affinity is described using a van’t Hoff-type relation:

9 $$K(T)={K}_{0}\exp \left(-\frac{\Delta H}{R}\left(\frac{1}{T}-\frac{1}{{T}_{0}}\right)\right),$$

where *ΔH* is the adsorption enthalpy, *R* is the gas constant, and *T*_0_ is the reference temperature.

The Gibbs free energy is given by

10 $$\Delta G=-RT\ln K.$$

This thermodynamic treatment is intended to describe an enthalpy-driven adsorption case around a defined reference temperature. For negative *ΔH*, the model predicts decreasing affinity with increasing temperature, as expected for an exothermic adsorption process. However, this behavior should not be generalized to all biochemical interactions. Systems involving strong entropic contributions, hydrophobic effects, conformational changes, surface restructuring, or complex hydration effects may show weaker temperature dependence or even opposite trends. The present temperature analysis therefore provides a controlled thermodynamic scenario rather than a universal description of all GO–biomolecule interactions.

### Kinetic Model

Time-dependent adsorption dynamics are described using a first-order reversible kinetic model:

11 $$\frac{d\Theta }{dt}={k}_{{\rm{on}}}C(1-\Theta )-{k}_{{\rm{off}}}\Theta ,$$

where *k*_on_ and *k*_off_ are effective association and dissociation rate constants.

The equilibrium constant is

12 $$K=\frac{{k}_{{\rm{on}}}}{{k}_{{\rm{off}}}}.$$

Characteristic times are defined as:

13 $${\tau }_{{\rm{assoc}}}=\frac{1}{{k}_{{\rm{on}}}C+{k}_{{\rm{off}}}},\quad {\tau }_{{\rm{diss}}}=\frac{1}{{k}_{{\rm{off}}}}.$$

The single-rate kinetic model is used as an ideal reversible limiting case. It is appropriate when adsorption and desorption can be approximated by one dominant effective binding population and when mass-transport, site heterogeneity, partial irreversibility, and slow surface rearrangements are neglected. Real GO interfaces may contain heterogeneous adsorption sites and may exhibit multi-rate or partially irreversible desorption. Therefore, the extracted kinetic constants should be interpreted as effective parameters of the assumed model rather than unique microscopic rate constants. This limitation is explicitly revisited in the Results section when discussing possible heterogeneous desorption behavior.

### Performance Metrics

The sensing performance is evaluated using standard SPR metrics. The resonance angle ${\theta }_{{\rm{res}}}$, minimum reflectance ${R}_{\min }$, and FWHM are extracted from the reflectance curves.

Detection accuracy is defined as

14 $$DA=\frac{1}{{\rm{FWHM}}}.$$

Sensitivity is evaluated as

15 $${S}_{C}=\frac{\Delta {\theta }_{{\rm{res}}}}{\Delta C},\quad {S}_{n}=\frac{\Delta {\theta }_{{\rm{res}}}}{\Delta n}.$$

A practical figure of merit (PFOM) is defined as

16 $$PFOM=\frac{1}{\sqrt{{R}_{\min }}\times {\rm{FWHM}}}.$$

The limit of detection is estimated as

17 $$LOD=\frac{\Delta {\theta }_{\min }}{{S}_{C}}.$$

Here, $\Delta {\theta }_{\min }$ is the assumed angular noise or angular resolution of the interrogation system. Consequently, the LOD reported by the model is not a unique experimental detection limit, but a theoretical resolution-dependent estimate. In the revised analysis, several angular-noise assumptions are considered to show explicitly how the predicted LOD scales with instrumental resolution.

Finally, the evanescent field penetration depth is

18 $${\delta }_{p}=\frac{1}{\Im ({k}_{z,s})}.$$

The reported sensitivities, PFOM values, and LOD estimates should therefore be interpreted as model-derived optical performance indicators under the stated assumptions. Experimental implementation would require calibration against real sensor noise, baseline drift, nonspecific background, surface preparation variability, and mass-transport conditions.

### Monte Carlo Robustness Analysis

To assess the sensitivity of the optimized SPR design to fabrication tolerances, we performed a Monte Carlo uncertainty analysis on the multilayer thicknesses. In each trial, the TiW, Au, and GO thicknesses were perturbed independently around their optimized values using uniformly distributed relative fluctuations:

19 $${d}_{i}^{(k)}={d}_{i,0}\left[1+{\delta }_{i}^{(k)}\right],$$

where *d*_*i*,0_ is the optimized thickness of layer *i* ∈ {TiW, Au, GO}, and ${\delta }_{i}^{(k)}$ is sampled from a zero-mean bounded interval representing the fabrication tolerance of that layer.

For each Monte Carlo realization, the perturbed stack was inserted into the transfer-matrix model and the resonance angle, minimum reflectance, linewidth, quality factor, and practical figure of merit were recalculated. Because PFOM contains $1/\sqrt{{R}_{\min }}$, rare realizations that approach critical coupling can produce very large PFOM values. The robustness analysis therefore emphasizes the directly extracted resonance quantities ${\theta }_{{\rm{res}}}$, ${R}_{\min }$, and FWHM. The resulting ensemble of outputs provides a numerical estimate of the robustness of the optimized design against thickness variations. In this work, the Monte Carlo analysis was applied to the final optimized stack using *n*_MC_ realizations at a representative analyte concentration and reference temperature.

Because the formal TiW and GO optima are subnanometer effective optical thicknesses, the Monte Carlo analysis should be interpreted as robustness around the formal optical optimum rather than as a complete fabrication-yield analysis of a physical GO monolayer. To address physical implementability separately, realistic GO/TiW cases are evaluated in the Results section using GO thicknesses in the 0.7–1.5 nm range and practical TiW adhesion-layer values.

## Results and Discussion

### Structural Comparison and Plasmonic Field Confinement

**Fig. 2:**
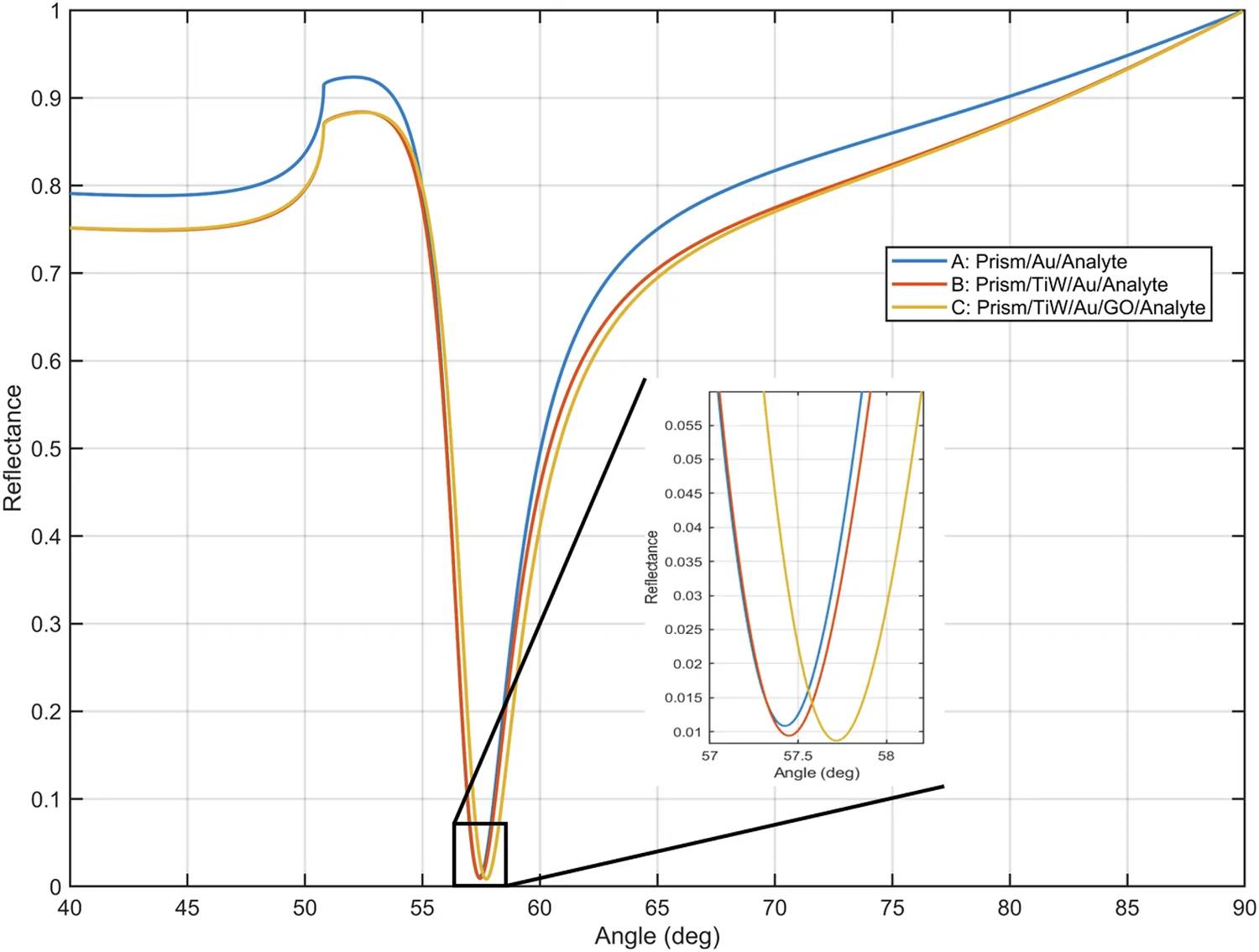
Angular reflectance spectra of the three SPR configurations at a representative concentration of 10 *μ*M. The progressive inclusion of TiW and GO layers leads to a deeper resonance dip and improved plasmonic coupling, while maintaining a nearly constant FWHM. The GO-containing configuration is interpreted as an adsorption-active SPR biointerface rather than as a fully specified capture-probe biosensor.The inset magnifies the resonance-minimum region

The sensing behavior of the proposed platform was first investigated by comparing three progressively engineered multilayer configurations, both at the architectural and optical-response levels, as shown in Figs. 1 and 2.

The corresponding angular reflectance spectra for each structure are shown in Fig. 2. At a representative concentration of 10 *μ*M and temperature of 25 ^∘^C, Structure A exhibits a resonance angle of 57.4248^∘^ with a minimum reflectance of 1.0836 × 10^−2^ and a full width at half maximum (FWHM) of 0.3719^∘^, yielding a PFOM of 25.830. Upon introducing the TiW layer (Structure B), the minimum reflectance decreases to 9.407 × 10^−3^, while the resonance angle shifts slightly to 57.4479^∘^ and the PFOM improves to 27.655. This indicates enhanced plasmonic coupling through improved interfacial matching at the prism–metal boundary.

The addition of the GO layer in Structure C further strengthens the optical response, leading to a minimum reflectance of 8.674 × 10^−3^ and a PFOM of 28.802, with a resonance angle of 57.7165^∘^. Importantly, the FWHM remains nearly constant across all three structures ( ~ 0.372^∘^), indicating that the performance improvement arises primarily from enhanced resonance depth rather than significant spectral narrowing.

To provide additional physical insight, field-confinement-related indicators were also evaluated. The resonance contrast increases from 92.28 in Structure A to 115.29 in Structure C, while the angular sharpness remains essentially unchanged. The resulting alternative figure of merit correspondingly increases from 248.14 to 309.25, confirming that the progressive inclusion of TiW and GO enhances plasmonic field confinement efficiency without compromising angular selectivity.

These results identify Structure C as the most suitable architecture for the subsequent adsorption-driven analysis. In the revised interpretation, the role of GO is not treated as a complete molecular-recognition layer by itself, but as an adsorption-active interfacial modifier that changes the local optical boundary condition and provides a surface where molecular accumulation can be represented through effective coverage. This distinction is important because no explicit antibody, aptamer, ligand, or DNA capture probe is introduced in the present model.

### Layer Optimization and Effective Interfacial Thickness Interpretation

**Fig. 3:**
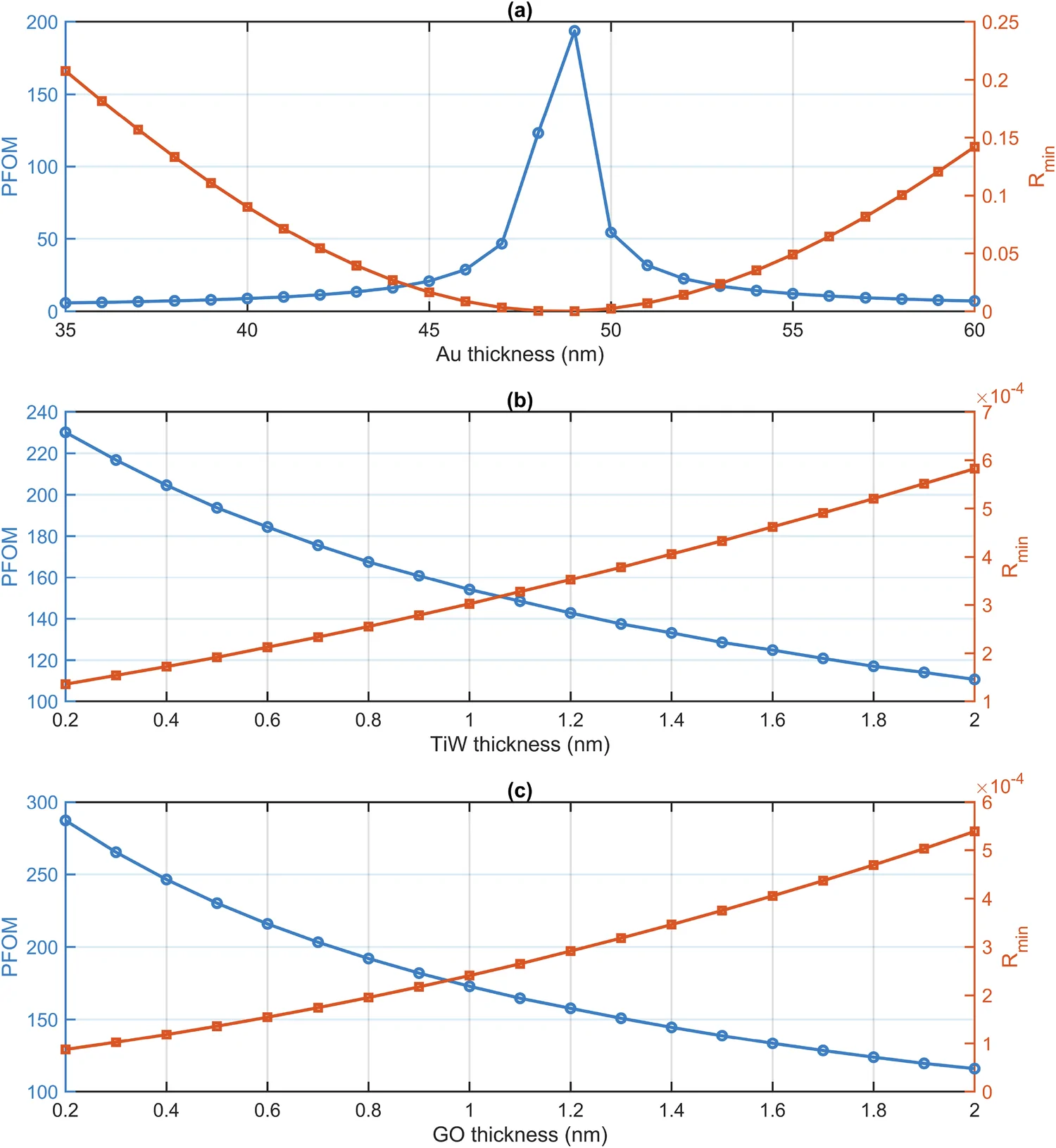
Layer thickness optimization at 10 *μ*M and 25 ^∘^C: (**a**) Au (PFOM${}_{\max }\approx 194$ at 49 nm), (**b**) TiW (PFOM${}_{\max }\approx 230$ at 0.20 nm), (**c**) GO (PFOM${}_{\max }\approx 287$ at 0.20 nm). The TiW and GO optima are interpreted as effective optical lower-bound thicknesses within the TMM calculation, not as literal physical monolayer thicknesses

Following the structural comparison, a systematic optimization of the multilayer architecture was performed in order to maximize plasmonic coupling efficiency and sensing performance of the complete Structure C. The combined optimization results for the Au, TiW, and GO layers are presented in Fig. 3.

The dependence of the sensor response on the gold thickness shows a clear optimum at 49.00 nm, where the practical figure of merit (PFOM) reaches 193.693, with a resonance angle of 57.6914^∘^, a minimum reflectance of 1.92 × 10^−4^, and a FWHM of 0.3729^∘^. This confirms that the gold film thickness primarily governs the plasmonic coupling condition.

The TiW adhesion layer exhibits a formal optical optimum at 0.20 nm, where the PFOM increases to 230.140 and the minimum reflectance decreases to 1.36 × 10^−4^. Similarly, the GO layer exhibits a formal optical optimum at 0.20 nm, where the PFOM reaches 287.130 and the minimum reflectance decreases to 8.8 × 10^−5^. These subnanometer values should not be interpreted as literal continuous physical layers or as the physical thickness of a GO monolayer. Instead, they represent lower-bound effective optical thicknesses within the transfer-matrix model, corresponding to the perturbative contribution of an ultrathin interfacial layer.

**Fig. 4:**
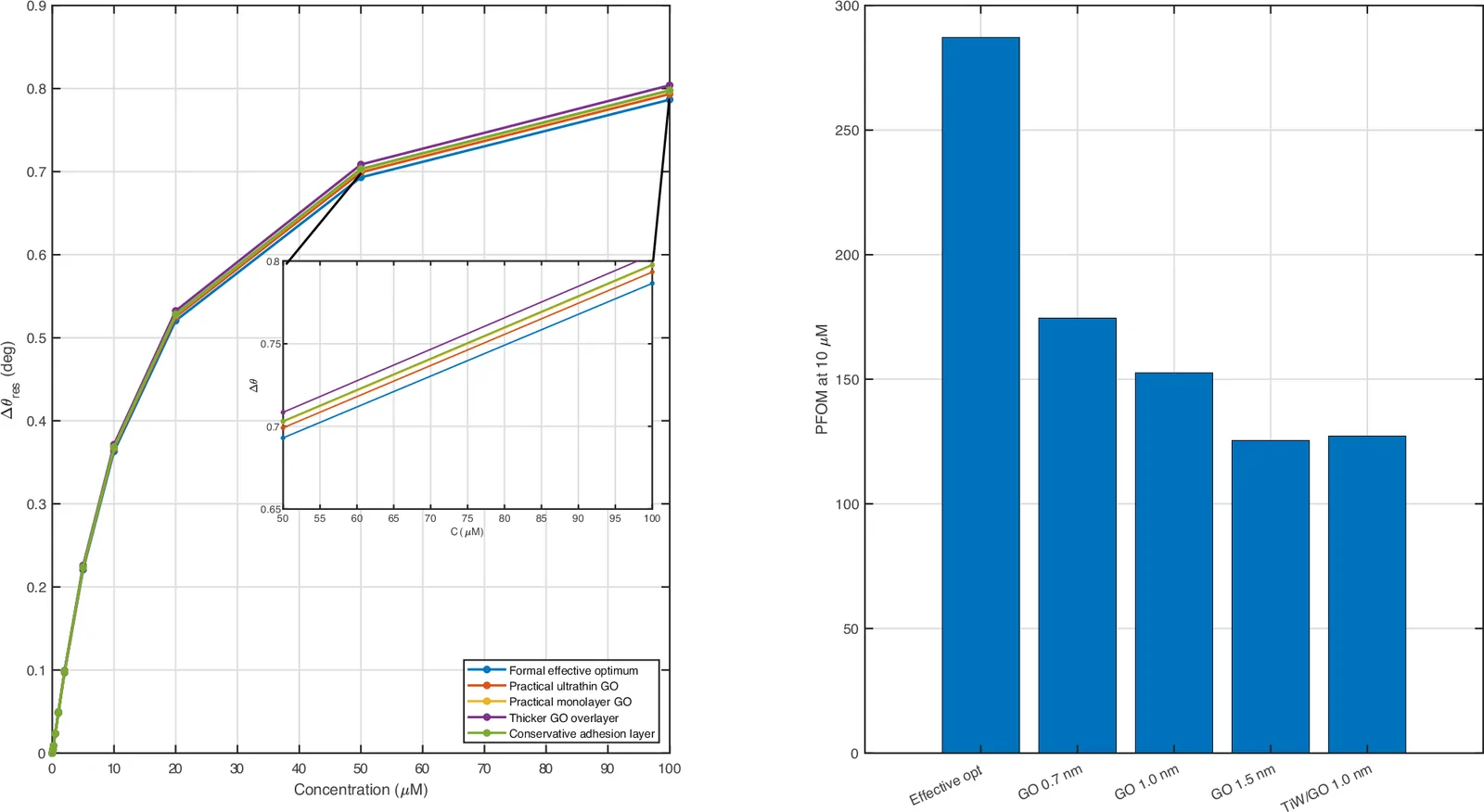
Comparison between the formal effective optical optimum and physically realistic GO/TiW thickness cases. **a** Resonance-angle shift, $\Delta {\theta }_{{\rm{res}}}$, as a function of analyte concentration for the different structural cases, showing that the adsorption-induced response is preserved for physically realistic GO/TiW thicknesses. **b** Practical figure of merit (PFOM) at 10 *μ*M for the same cases, indicating that although the formal effective optimum gives the highest PFOM, physically realistic GO/TiW configurations retain comparable sensing performance

This interpretation is necessary because real GO monolayers are generally expected to have larger apparent physical thicknesses depending on oxidation degree, hydration, substrate interaction, and measurement method. The optimization result therefore indicates that plasmonic coupling favors the weakest lossy GO/TiW perturbation compatible with an adsorption-active interface, rather than proving that a 0.20 nm physical GO film should be fabricated. It is also worth noting that subnanometer TiW adhesion layers have precedent in recent theoretical multilayer SPR modeling; for example, a related GO/TiW/Au SPR design reported a TiW adhesion layer of 0.2 nm together with a 46 nm Au film and a 0.5 nm GO layer [31]. In the present work, this value is therefore used as a formal effective optical lower-bound in the TMM optimization, while physically more realistic GO/TiW cases are evaluated separately in Fig. 4 and Table 2.

**Table 2:** Effective and physically realistic GO/TiW thickness cases evaluated at 10 *μ*M

Case	TiW (nm)	GO (nm)	Shift at 10 *μ*M (deg)	*S*_*C*_ (deg *μ*M^−1^)	LOD (*μ*M)
Formal effective optimum	0.20	0.20	0.3632	0.03708	0.02697
Practical ultrathin GO	0.50	0.70	0.3664	0.03741	0.02673
Practical monolayer GO	0.50	1.00	0.3683	0.03760	0.02659
Thicker GO overlayer	0.50	1.50	0.3714	0.03792	0.02637
Conservative adhesion layer	1.00	1.00	0.3686	0.03763	0.02657

The formal optimum maximizes PFOM, while realistic GO/TiW cases preserve comparable concentration sensitivity

To test whether the concentration-dependent response survives under physically more realistic interfacial thicknesses, additional simulations were performed using GO thicknesses of 0.70, 1.00, and 1.50 nm, together with practical TiW values of 0.50 or 1.00 nm. The results are shown in Fig. 4 and summarized in Table 2. Although the formal effective optimum provides the largest PFOM because of its very deep reflectance minimum, all realistic GO/TiW cases preserve nearly the same adsorption-driven response. In particular, the low-concentration sensitivity remains close to 0.037–0.038 deg *μ*M^−1^, and the model-estimated LOD at an angular resolution of 10^−3^ degree remains close to 0.027*μ*M.

### Adsorption-driven Concentration Response

**Fig. 5:**
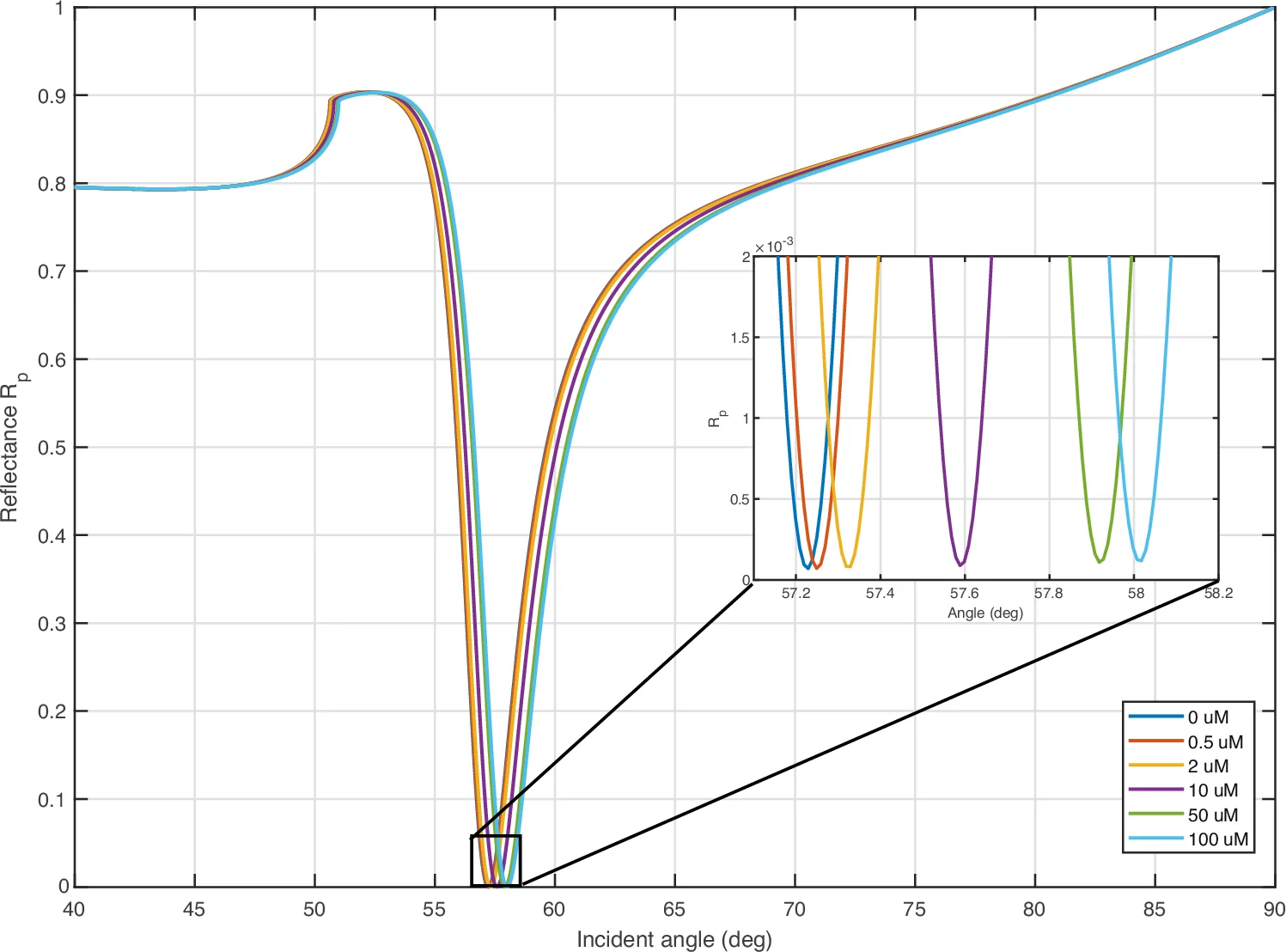
Angular reflectance spectra of the optimized multilayer SPR biointerface for increasing analyte concentrations from 0 to 100 *μ*M. The resonance dip shifts monotonically toward higher angles, confirming adsorption-driven modulation of the interfacial refractive index. The response is interpreted through effective surface coverage and optically equivalent adlayer formation. The inset magnifies the resonance-shift region

The concentration-dependent response of the optimized multilayer SPR biointerface was investigated by explicitly coupling the optical response to adsorption-governed surface coverage. The resulting angular reflectance spectra are shown in Fig. 5. As the analyte concentration increases from 0 to 100 *μ*M, the resonance dip shifts monotonically toward higher incidence angles, reflecting the progressive modification of the interfacial optical environment induced by molecular accumulation at the GO surface.

At zero concentration, the optimized effective stack exhibits a resonance angle of 57.2271^∘^ with a minimum reflectance of 6.9 × 10^−5^ and a FWHM of 0.3715^∘^. As the concentration increases to 100 *μ*M, the resonance angle reaches 58.0136^∘^, corresponding to a total angular shift of 0.7865^∘^. Over the same range, the minimum reflectance increases moderately to 1.13 × 10^−4^, while the FWHM remains nearly constant, varying only between 0.3710^∘^ and 0.3725^∘^. Consequently, the Q-factor remains stable in the range 153.753–156.189, indicating that adsorption primarily affects the resonance position without strongly degrading angular sharpness.

The monotonic angular shift confirms that the simulated SPR response is governed by adsorption-induced interfacial perturbations. As the analyte concentration increases, the effective refractive index of the sensing region rises from 1.332000 to 1.338955, while the surface coverage evolves from *Θ* = 0 to *Θ* = 0.9078. This produces an effective biolayer thickness increasing up to 2.7235 nm.

The quantity *d*_bio_ should be interpreted as an optically equivalent hydrated adlayer thickness rather than the geometric size of a specified molecule. It represents the averaged optical contribution of adsorbed material, molecular orientation, packing density, hydration, and local interfacial organization. In the main moderate-adsorption case, ${d}_{{\rm{bio}}}={d}_{\max }\Theta$ with ${d}_{\max }=3$ nm, giving *d*_bio_ = 2.7235 nm at the highest simulated coverage. This value is therefore an effective optical parameter consistent with a thin hydrated adsorbed layer, not a claim that a particular biomolecule has this physical thickness.

A key feature of the response is its nonlinear behavior, arising from the finite number of adsorption sites available at the interface. The local sensitivity is highest in the low-concentration regime, reaching a maximum value of 0.06005 deg *μ*M^−1^ at approximately 0.10 *μ*M. As the concentration increases, the sensitivity gradually decreases due to progressive saturation of the adsorption sites, marking the transition from a quasi-linear regime to a saturation-dominated regime consistent with Langmuir–Hill adsorption behavior.

These results establish an effective physical mapping from concentration to coverage, from coverage to interfacial optical perturbation, and from interfacial perturbation to SPR shift. The mapping should be understood as an adsorption-driven biointerface model rather than as a complete model of specific molecular recognition.

concentration  → surface coverage  → effective interfacial optical perturbation  → SPR shift.

### Calibration Characteristics, Effective Detection Limits, and Adlayer Interpretation

**Fig. 6:**
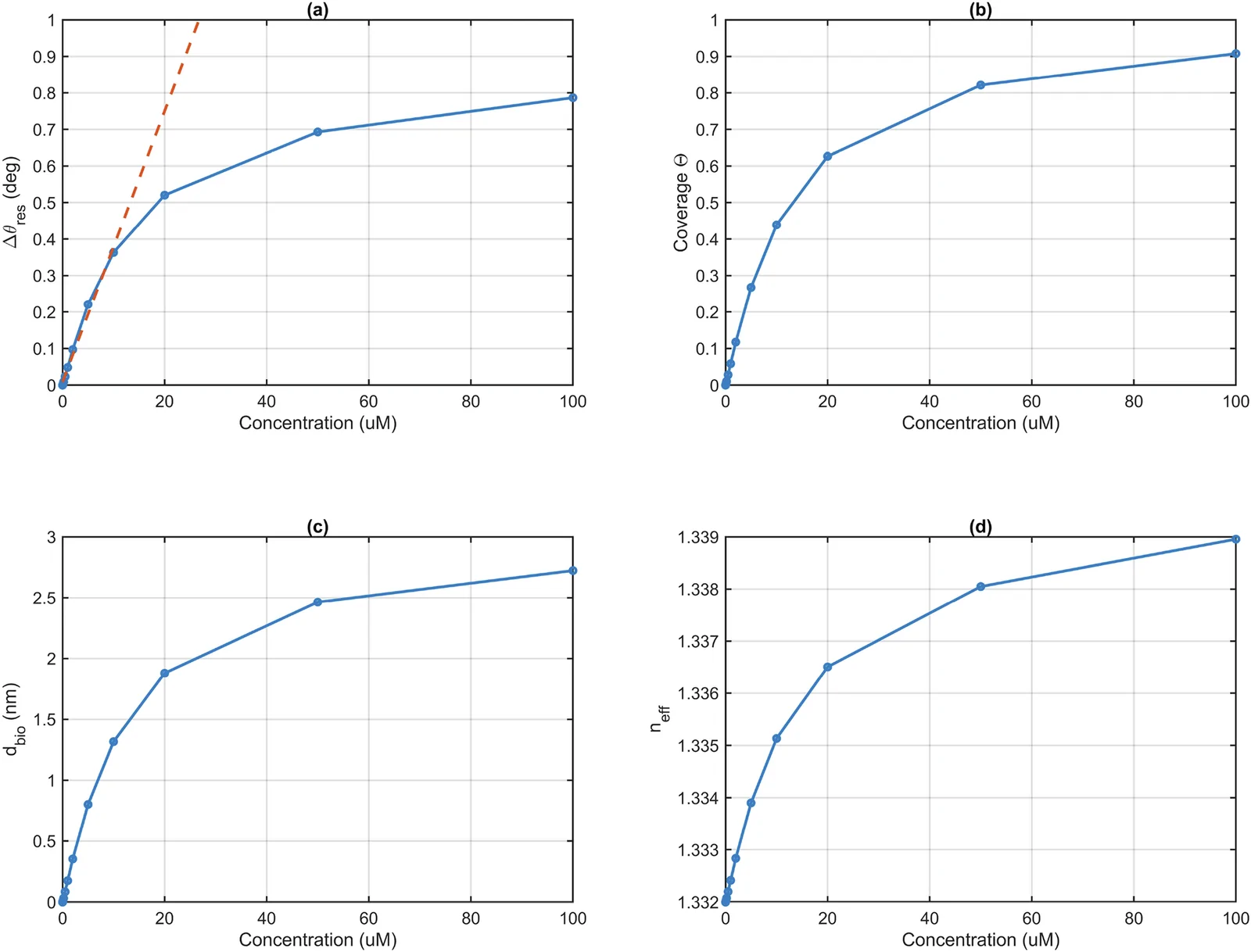
Calibration and adsorption characteristics of the optimized SPR biointerface. **a** Resonance-angle shift as a function of analyte concentration with linear fit in the low-concentration regime. **b** Surface coverage following Langmuir–Hill adsorption behavior. **c** Effective biolayer thickness as a function of concentration. **d** Effective refractive index of the sensing region. The biolayer thickness is interpreted as an optically equivalent hydrated adlayer

The quantitative sensing performance of the optimized SPR biointerface was evaluated through calibration analysis, adsorption behavior, and interfacial optical evolution, as summarized in Fig. 6.

The calibration curve, shown in Fig. 6a, exhibits a clear monotonic relationship between analyte concentration and resonance-angle shift. In the low-concentration regime (0–10 *μ*M), linear regression yields a sensitivity of 0.037081 deg *μ*M^−1^ with a coefficient of determination *R*^2^ = 0.988270. This regime corresponds to partial occupation of adsorption sites, where the response remains approximately linear before saturation effects become dominant.

The corresponding refractive-index sensitivity, extracted from the effective index variation, reaches 115.963 deg RIU^−1^ with *R*^2^ = 0.999999. Based on an angular resolution of 10^−3^ degree, the theoretical limits of detection are estimated as 0.026968*μ*M in concentration and 8.62 × 10^−6^ RIU.

These LOD values are not experimental detection limits. They are model-estimated values obtained under an assumed angular resolution. To make this dependence explicit, the LOD was recalculated for angular noise/resolution values ranging from 10^−4^ to 10^−2^ degree (Table 3). The concentration LOD scales from 0.0027*μ*M to 0.270*μ*M across this range, demonstrating that the detection limit should be interpreted as instrument-resolution-dependent rather than as an intrinsic property of the GO layer alone.

**Table 3:** Noise-dependent theoretical limit of detection

Angular noise/resolution (deg)	LOD (*μ*M)	LOD (RIU)
10^−4^	0.0027	8.62 × 10^−7^
5 × 10^−4^	0.0135	4.31 × 10^−6^
10^−3^	0.0270	8.62 × 10^−6^
5 × 10^−3^	0.1348	4.31 × 10^−5^
10^−2^	0.2697	8.62 × 10^−5^

The values are model estimates and scale directly with the assumed angular resolution

The adsorption behavior is presented in Fig. 6b, where the surface coverage follows a characteristic Langmuir–Hill profile. The coverage increases from *Θ* = 0 to *Θ* = 0.9078, indicating that the system approaches, but does not fully reach, saturation within the investigated concentration range. From this curve, the half-coverage concentration is estimated to be on the order of 10*μ*M, consistent with the nominal moderate-regime value *K*_*D*_ = 1/*K* ≈ 12.5*μ*M.

The Hill coefficient is approximately *n* ≈ 1.10, indicating weak positive cooperativity and a predominantly non-cooperative adsorption response in the moderate regime. Figures 6c and 6d show that the effective biolayer thickness and effective refractive index increase monotonically with concentration. The combined interpretation is therefore not that a known biomolecule of fixed geometry forms a precisely defined layer, but that the optical response can be represented by an effective hydrated adlayer and a coverage-dependent refractive-index perturbation.

Taken together, the calibration and adsorption analysis demonstrates a quantitative link between adsorption parameters and optical response under the assumptions of the model. The revised interpretation avoids treating the simulated platform as an experimentally validated or universally specific biosensor. Instead, it establishes a computational relationship between GO-mediated surface accumulation, effective optical layer formation, and SPR angular readout.

### Temperature-dependent Thermodynamic Behavior

**Fig. 7:**
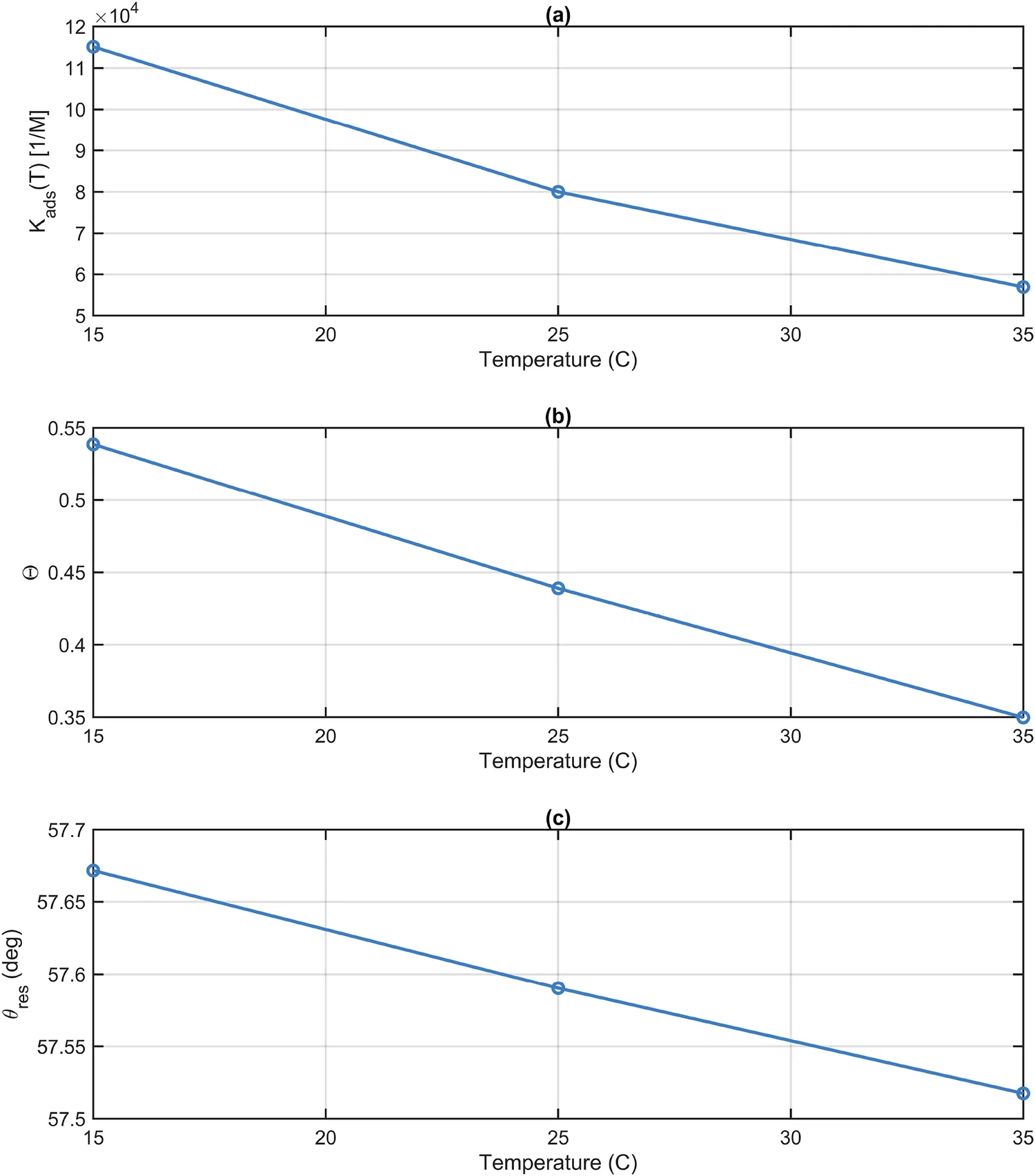
Temperature-dependent behavior of the optimized SPR biointerface at a representative concentration of 10 *μ*M. **a** Variation of the adsorption affinity constant *K*(*T*) with temperature. **b** Corresponding surface coverage *Θ*. **c** Resulting resonance angle shift. The decreasing affinity with temperature corresponds to the assumed enthalpy-driven exothermic adsorption case

The influence of temperature on the adsorption-driven SPR response was investigated in order to assess the thermodynamic consistency of the proposed biointerface model. The results, obtained for a representative analyte concentration of 10 *μ*M, are summarized in Fig. 7.

As the temperature increases from 15 ^∘^C to 35 ^∘^C, a systematic decrease in adsorption affinity is observed. The equilibrium adsorption constant decreases from 1.1513 × 10^5^ M^−1^ at 15 ^∘^C to 5.6920 × 10^4^ M^−1^ at 35 ^∘^C. This reduction in affinity leads to a corresponding decrease in surface coverage, from *Θ* = 0.53866 at 15 ^∘^C to *Θ* = 0.34981 at 35 ^∘^C.

The decrease in surface coverage directly affects the optical response. The resonance angle shifts from 57.6715^∘^ at 15 ^∘^C to 57.5175^∘^ at 35 ^∘^C, indicating that higher temperatures reduce the effective refractive-index perturbation at the sensing interface under the chosen thermodynamic parameters.

The temperature dependence of the adsorption process is interpreted using the van’t Hoff relation. A linear fit of *łn K* as a function of inverse temperature yields an estimated adsorption enthalpy of *ΔH* ≈ − 25.97 kJ mol^−1^. The negative value of *ΔH* is consistent with an exothermic adsorption process, which explains the observed decrease in binding affinity with increasing temperature.

This conclusion is specific to the enthalpy-driven exothermic case used in the simulations. It should not be generalized to all biochemical interactions. In systems involving hydrophobic effects, conformational changes, dehydration, or entropy-dominated binding, the temperature dependence may be weaker or may even show the opposite trend. For the main model at 25 ^∘^C, the calculated Gibbs free energy is *ΔG* ≈ − 27.99 kJ mol^−1^, while *ΔH* = − 26 kJ mol^−1^, corresponding to a small implicit entropy contribution. The present temperature analysis therefore provides a controlled thermodynamic example rather than a universal prediction for GO biointerfaces.

### Kinetic Response and Dynamic Adsorption Behavior

**Fig. 8:**
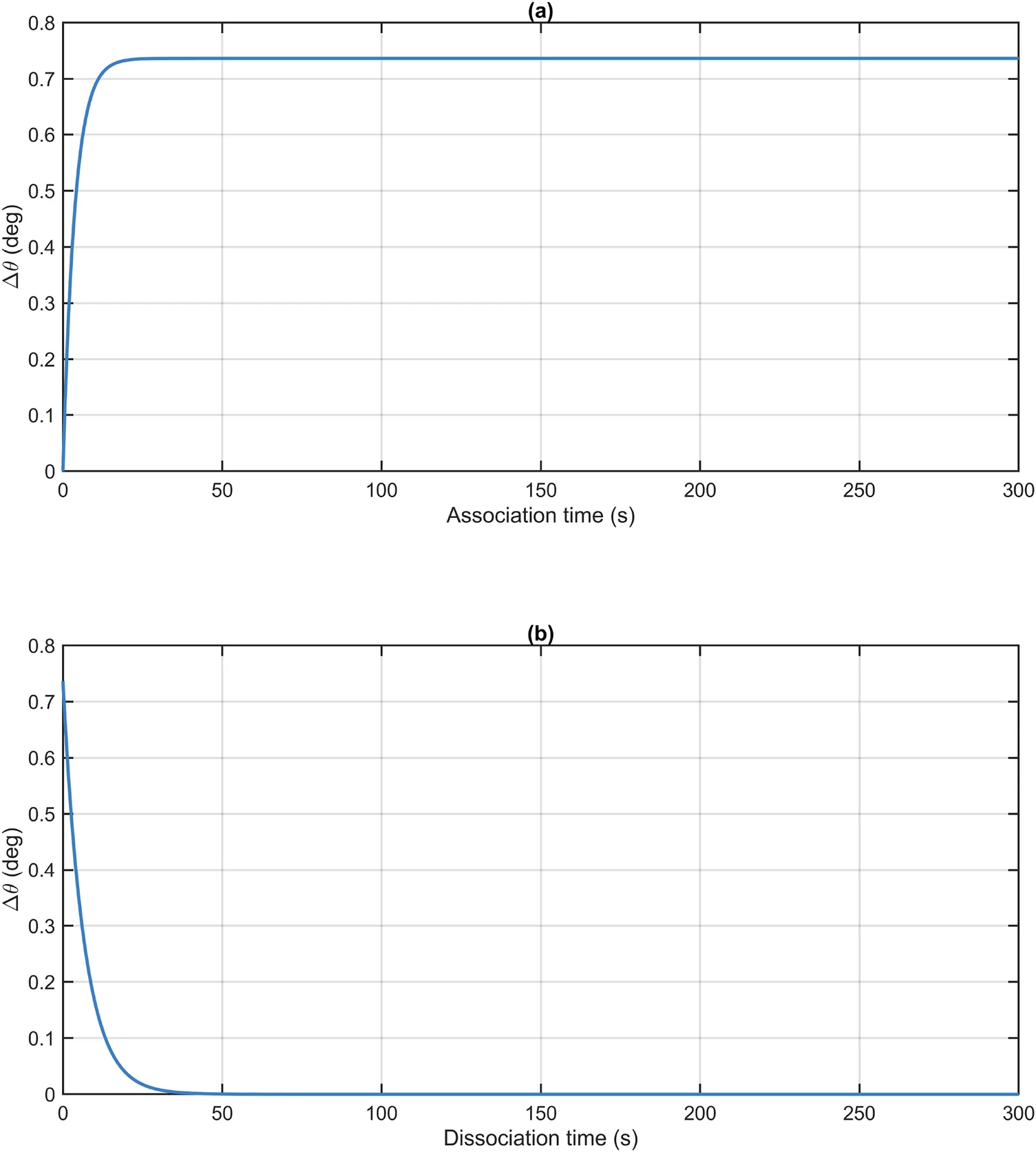
Simulated kinetic response of the optimized SPR biointerface at 10 *μ*M analyte concentration. **a** Association phase showing the buildup of surface coverage and resonance shift. **b** Dissociation phase showing exponential decay after analyte removal. The curves represent an ideal reversible single-rate limiting case for adsorption dynamics

To further extend the analysis beyond equilibrium conditions, the time-dependent adsorption dynamics of the optimized SPR biointerface were investigated using a first-order kinetic model. The association and dissociation processes were simulated for a representative concentration of 10 *μ*M, and the resulting sensorgrams are shown in Fig. 8.

During the association phase, the surface coverage increases progressively as molecules accumulate on available effective sites at the GO interface. This results in a corresponding increase in the resonance-angle shift, which approaches a steady-state plateau as equilibrium is reached. The characteristic association time constant is estimated as *τ*_assoc_ = 3.704 s under the chosen parameter set.

Upon removal of the analyte, the dissociation phase is modeled as an exponential decay governed by *k*_off_. The corresponding optical response follows the same trend, with the resonance angle gradually returning toward its baseline value. The dissociation time constant is *τ*_diss_ = 6.667 s.

The equilibrium surface coverage predicted by the kinetic model is *Θ*_eq_ = 0.43894, in agreement with the steady-state adsorption analysis. From the kinetic parameters, the effective dissociation constant is *K*_*D*_ = *k*_off_/*k*_on_ ≈ 1.25 × 10^−5^ M, consistent with the moderate adsorption regime.

The single-exponential association–dissociation model is retained as the simplest reversible first-order limiting case. This assumption is appropriate when the effective adsorption sites are treated as homogeneous and when transport and rebinding effects are not explicitly resolved. Real GO surfaces, however, are chemically heterogeneous and may exhibit multiple adsorption environments, partially irreversible binding, or multi-rate desorption. In such cases, heterogeneous desorption models with fast and slow site populations could generate long dissociation tails. These extensions are beyond the scope of the present single-rate framework, but define a clear direction for future experimental fitting.

### Parametric Adsorption Regimes and Multi-regime Response

The multi-regime analysis was revised to clarify that the weak, moderate, and strong cooperative cases are parametric adsorption regimes rather than assigned real biomolecules. Their purpose is to explore how affinity and cooperativity affect the optical response of the same GO-SPR interface. The corresponding affinity constants, equivalent dissociation constants, Hill coefficients, and possible physical interpretations are summarized in Table 4.

**Table 4:** Parametric adsorption regimes used in the multi-regime analysis

Regime	*K*_25_ (M^−1^)	*K*_*D*_ (*μ*M)	Hill *n*	Interpretation
Weak binder	5.0 × 10^3^	200	1.00	Weak nonspecific adsorption
Moderate binder	8.0 × 10^4^	12.5	1.10	Moderate GO accumulation
Strong cooperative binder	6.0 × 10^5^	1.67	1.70	Multivalent/cooperative accumulation

The categories are not assigned to specific biomolecules, but represent effective GO-surface accumulation regimes

**Fig. 9:**
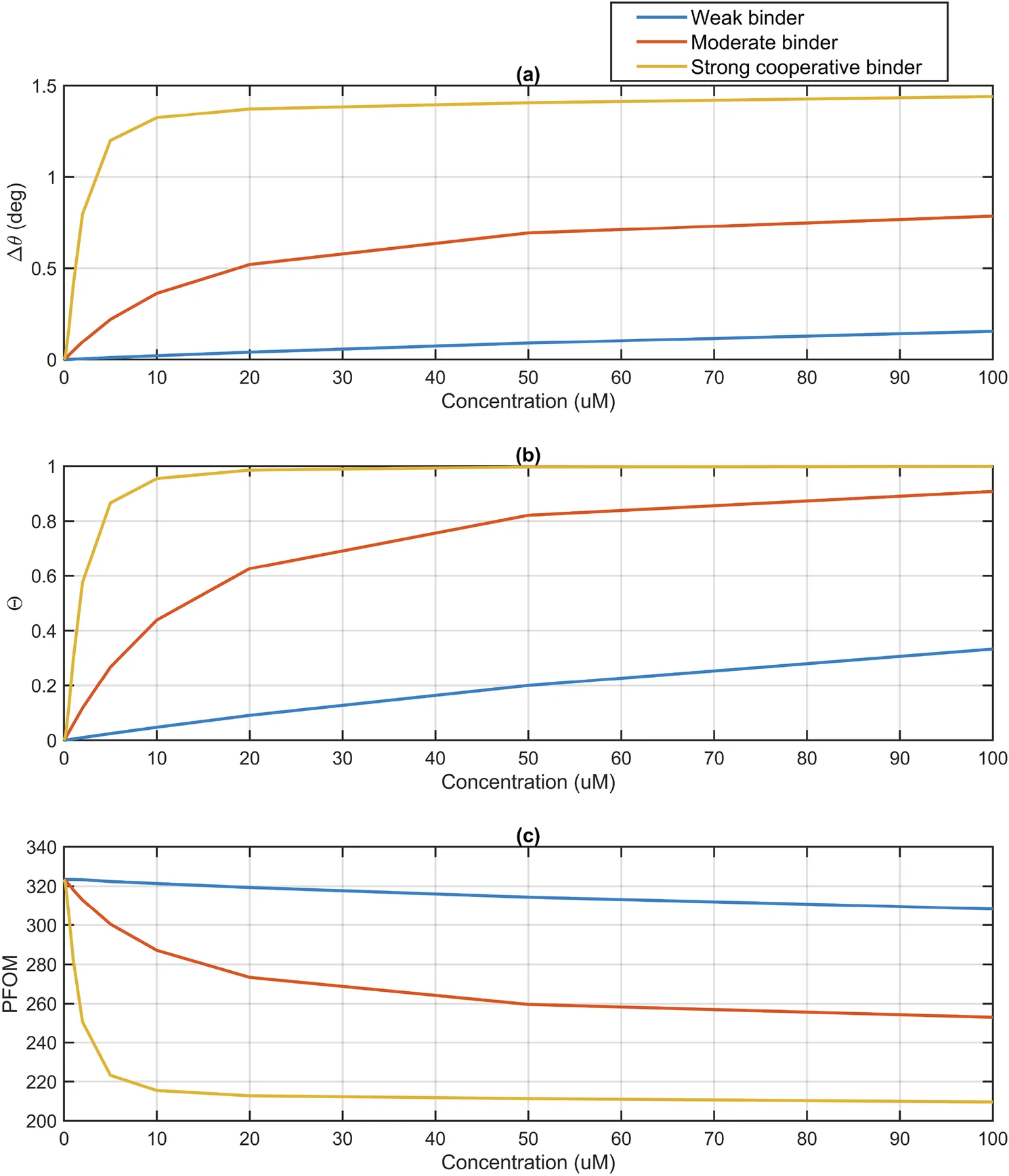
Multi-regime comparison of the optimized SPR biointerface. **a** Resonance angle shift as a function of concentration for weak, moderate, and strong cooperative adsorption regimes. **b** Corresponding surface coverage curves illustrating distinct adsorption behavior. **c** Practical figure of merit (PFOM). The three cases are parametric adsorption regimes, not assigned molecular species

The comparative results are presented in Fig. 9. The weak binder exhibits a nearly linear response over the concentration range, reflecting low surface occupancy and minimal saturation effects. The moderate binder shows a nonlinear but broadly distributed response, with a gradual transition from linear to saturation regimes. The strong cooperative binder displays a much steeper response at intermediate concentrations, characteristic of cooperative adsorption, followed by rapid saturation at higher concentrations.

These trends are confirmed by the surface coverage curves. The weak binder reaches only moderate coverage even at high concentration, while the moderate binder achieves intermediate coverage levels with a smooth Langmuir-like profile. The strong cooperative binder exhibits a sigmoidal adsorption curve due to its Hill coefficient (*n* = 1.7), reflecting cooperative surface accumulation.

The PFOM analysis provides additional insight into the practical sensing response. At 10 *μ*M, the weak regime yields the highest PFOM value (321.321), followed by the moderate regime (287.130) and the strong cooperative regime (215.496). This trend occurs because strong cooperative accumulation produces larger perturbations and faster saturation, which can reduce resonance contrast and practical detectability. Thus, stronger affinity does not automatically imply better optical performance.

This analysis should therefore be interpreted as a parametric study of adsorption regimes rather than as a demonstration of selectivity among real analytes. The model can distinguish response shapes associated with different affinity/cooperativity parameters, but experimental assignment to specific biomolecules would require defined surface chemistry, capture probes if selectivity is required, and calibration against measured sensorgrams.

### Monte Carlo Robustness of the Effective Optimized Stack

**Fig. 10:**
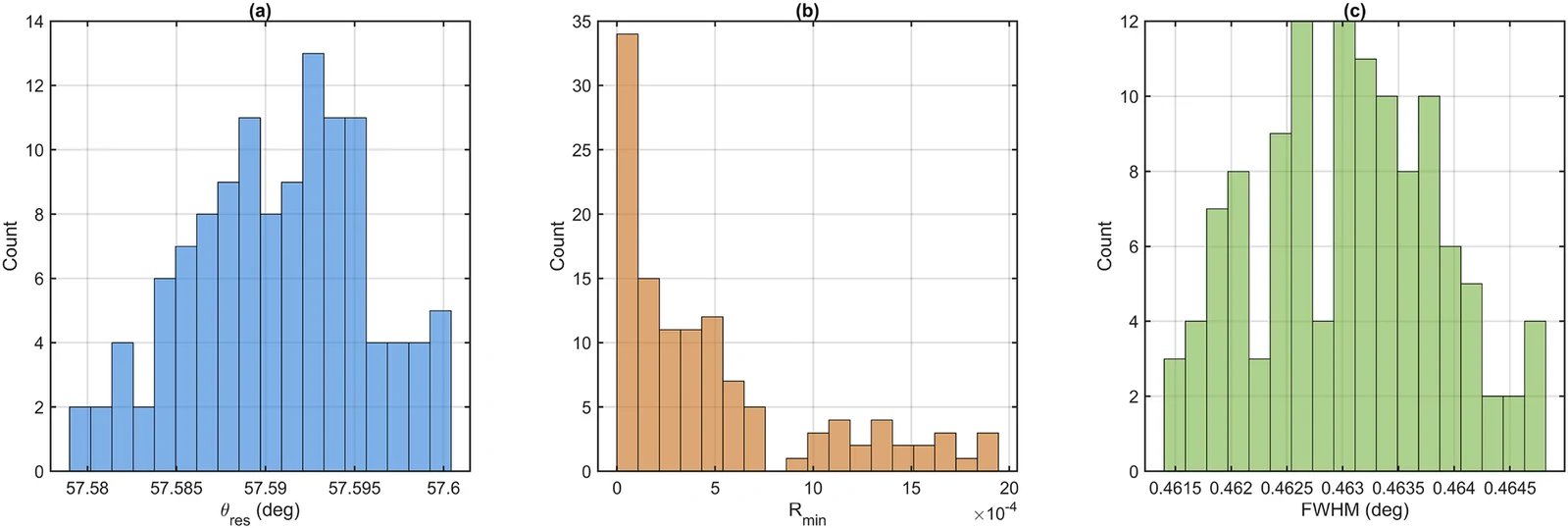
Monte Carlo robustness analysis of the optimized Prism/TiW/Au/GO effective stack at 10 *μ*M and 25 ^∘^C. **a** Distribution of resonance angle ${\theta }_{{\rm{res}}}$. **b** Distribution of minimum reflectance ${R}_{\min }$. **c** Distribution of linewidth FWHM. The analysis tests numerical stability around the formal effective optimum

Figure 10 summarizes the Monte Carlo propagation of thickness uncertainty for the optimized Prism/TiW/Au/GO effective stack at 10 *μ*M and 25 ^∘^C. The optimized geometry was sampled over 120 realizations by perturbing the TiW, Au, and GO effective thicknesses within  ± 10%,  ± 2%, and  ± 10%, respectively.

The resonance angle remains tightly distributed around approximately $\langle {\theta }_{{\rm{res}}}\rangle \approx 57.5{9}^{\circ }$, with a standard deviation of about 5 × 10^−3^ degree, demonstrating a narrow angular distribution across the sampled uncertainty window. The linewidth is also stable, remaining close to ⟨FWHM⟩ ≈ 0.463^∘^ with fluctuations on the order of 10^−3^ degree. The minimum reflectance remains low on average, although individual realizations can approach very deep minima because the simulated structure operates close to critical plasmonic coupling.

Because the PFOM depends inversely on $\sqrt{{R}_{\min }}$, rare realizations close to critical coupling can produce very large PFOM values. The robustness discussion therefore focuses on the directly extracted resonance quantities ${\theta }_{{\rm{res}}}$, ${R}_{\min }$, and FWHM.

Because this Monte Carlo analysis is centered on the formal effective optimum, it should be interpreted as a numerical robustness test of the optical design rather than as a fabrication claim for a literal 0.20 nm continuous GO layer. The realistic-thickness comparison in Fig. 4 provides the complementary physical-realism check. Together, the two analyses show that the formal optical optimum is numerically stable and that physically realistic GO/TiW cases preserve the adsorption-driven concentration response.

### Model Scope, Experimental Relevance, and Limitations

The revised results clarify the scope of the proposed GO-SPR framework. First, the model describes adsorption-driven interfacial accumulation and optical transduction, not a complete selective biosensor with a defined molecular-recognition chemistry. Specific biosensing applications such as antibody–antigen detection, aptamer recognition, ligand–receptor binding, or DNA hybridization would require explicit capture-probe functionalization and experimental calibration.

Second, the TiW and GO thicknesses obtained from formal optimization are effective optical parameters. The realistic-thickness simulations demonstrate that GO thicknesses in the 0.70–1.50 nm range preserve the concentration-dependent response, even though the formal lower-bound optimum gives a higher PFOM. Third, the effective biolayer thickness is an optically equivalent hydrated adlayer parameter, not the physical size of a specified analyte. Fourth, the estimated LOD is instrument-resolution-dependent and should not be interpreted as an experimentally validated detection limit. Fifth, the single-rate kinetic model and the enthalpy-driven temperature dependence are idealized limiting cases that provide mechanistic insight but do not capture all possible heterogeneous, partially irreversible, or entropy-dominated GO adsorption processes.

From an experimental perspective, the predicted angular sensitivity of 115.96 deg RIU^−1^ and the noise-dependent RIU-scale LOD are within the range expected for angular-interrogation SPR simulations, but they should be treated as theoretical estimates because no experimental noise, drift, surface-preparation variability, or real analyte calibration is included. Experimental implementation would require fabrication of the Au/TiW/GO stack, independent characterization of GO thickness and coverage, and calibration using measured adsorption sensorgrams under controlled buffer, temperature, and flow conditions.

Within these limits, the model provides a physically interpretable computational framework for linking adsorption thermodynamics, kinetic parameters, effective interfacial optical properties, and SPR resonance shifts. Its main utility is therefore in mechanism-aware design and interpretation of GO-modified SPR biointerfaces, rather than in replacing experimental validation for a specific biochemical sensing system.

## Conclusion

In this work, an adsorption-driven theoretical framework was developed for graphene oxide (GO)-functionalized multilayer SPR biointerfaces. The model combines transfer-matrix optical calculations with generalized Langmuir–Hill adsorption, thermodynamic analysis, and first-order kinetic modeling to connect analyte concentration, effective surface coverage, interfacial optical perturbation, and SPR resonance response. The revised interpretation explicitly treats the system as an effective GO-mediated adsorption biointerface rather than as a complete capture-probe biosensor.

The optical analysis shows that the Prism/TiW/Au/GO configuration provides stronger plasmonic coupling than the corresponding Prism/Au and Prism/TiW/Au structures. Formal optimization of the multilayer stack gives an Au thickness of 49 nm and lower-bound effective TiW and GO optical thicknesses of 0.20 nm. These subnanometer TiW and GO values are interpreted as effective optical parameters within the transfer-matrix model, not as literal continuous physical monolayers. To address physical realism, additional simulations using GO thicknesses of 0.70–1.50 nm and practical TiW values were included. These cases preserve the adsorption-driven concentration response and maintain low-concentration sensitivities close to 0.037–0.038 deg *μ*M^−1^, confirming that the main transduction behavior is not restricted to the formal lower-bound optimum.

For the moderate adsorption regime, the model predicts a monotonic resonance shift of approximately 0.79^∘^ over the 0–100 *μ*M concentration range, together with a refractive-index sensitivity of 115.96 deg RIU^−1^. The effective biolayer thickness reaches 2.72 nm at high coverage; this value is interpreted as an optically equivalent hydrated adlayer that averages over adsorbed material, hydration, molecular orientation, packing, and dielectric contrast, rather than as the physical size of a specified molecule. The calibration analysis further shows that the theoretical LOD depends directly on the assumed angular noise. For an angular resolution of 10^−3^ degree, the estimated LOD is 0.027*μ*M, while varying the angular noise from 10^−4^ to 10^−2^ degree gives LOD values from 0.0027 to 0.270*μ*M.

The thermodynamic analysis illustrates an enthalpy-driven exothermic adsorption case, with an estimated *ΔH* ≈ − 26 kJ mol^−1^, for which the adsorption affinity decreases with increasing temperature. This trend should not be generalized to all biochemical systems, since entropy-dominated binding, hydrophobic effects, conformational changes, and hydration effects may lead to different temperature responses. Similarly, the kinetic response is described using a reversible single-rate association–dissociation model, which provides an ideal limiting case for interpreting adsorption dynamics. Real GO interfaces may contain heterogeneous sites, slow rearrangements, rebinding, or partially irreversible adsorption, and the extracted kinetic constants should therefore be understood as effective model parameters.

The weak, moderate, and strong cooperative cases considered in this study should be interpreted as parametric adsorption regimes rather than assigned molecular species. The analysis shows that stronger affinity or stronger cooperativity does not automatically produce better optical performance, because rapid saturation and stronger interfacial perturbation can reduce practical detectability. This highlights the need to consider both adsorption behavior and plasmonic response when designing or interpreting GO-modified SPR interfaces.

Overall, the proposed framework provides a mechanism-aware computational approach for studying adsorption-driven GO-SPR biointerfaces. Its main contribution is the explicit connection between adsorption thermodynamics, kinetic response, effective interfacial optical-layer formation, and SPR angular transduction, while clearly identifying the limits of the effective-parameter description. Future experimental implementation would require defined surface preparation, realistic noise and drift characterization, analyte-specific calibration, and, for selective biosensing applications, explicit capture-probe functionalization. Within these limits, the model can guide the interpretation and design of GO-modified SPR interfaces for adsorption-dominated molecular accumulation and interfacial biophysics.

## Data Availability

The simulation data, numerical tables, and MATLAB code used to generate the results are available from the corresponding author upon reasonable request.
